# High-frequency hearing, tinnitus, and patient satisfaction with stapedotomy: A randomized prospective study

**DOI:** 10.1038/srep13341

**Published:** 2015-08-21

**Authors:** Dan Bagger-Sjöbäck, Karin Strömbäck, Malou Hultcrantz, Georgios Papatziamos, Henrik Smeds, Niklas Danckwardt-Lillieström, Bo Tideholm, Ann Johansson, Sten Hellström, Pierre Hakizimana, Anders Fridberger

**Affiliations:** 1 Department of Otolaryngology, Karolinska University Hospital, Stockholm, Sweden; 2 Department of Clinical Science, Intervention and Technology, Karolinska Institutet, Stockholm, Sweden; 3 Department of Surgical Sciences, Section of Otorhinolaryngology and Head and Neck Surgery, Uppsala University, Uppsala, Sweden; 4 Department of Audiology and Neurotology, Karolinska University Hospital, Stockholm, Sweden; 5 Department of Clinical and Experimental Medicine, Cell Biology, Linköping University, Linköping, Sweden

## Abstract

Otosclerosis is a common disorder that leads to conductive hearing loss. Most patients with otosclerosis also have tinnitus, and surgical treatment is known to improve hearing as well as tinnitus. Some patients however experience worsening of tinnitus after the operation, but there are no known factors that allow surgeons to predict who will be at risk. In this prospective observational study on 133 patients undergoing stapedotomy, we show that postoperative air conduction thresholds at very high stimulus frequencies predict improvement of tinnitus, as assessed with proportional odds logistic regression models. Young patients were significantly more likely to experience reduction of tinnitus and patients whose tinnitus became better were also more satisfied with the outcome of the operation. These findings have practical importance for patients and their surgeons. Young patients can be advised that surgery is likely to be beneficial for their tinnitus, but a less positive message should be conveyed to older patients.

Otosclerosis is a common disorder^1^,^2^, where one of the ossicles in the middle ear, the stapes, gradually fuses with surrounding bone, producing conductive hearing loss. This hearing impairment can be treated surgically^3^, commonly by replacing the diseased stapes with a prosthesis that improves sound transmission through the middle ear to the sensory cells in the inner ear. The usual result is great enhancement of low-frequency hearing^4^. At frequencies below 1000 Hz, the average improvement is around 30 dB, which substantially enhances speech perception and frequently means patients no longer need hearing aids.

Despite being a generally successful procedure that improves hearing in a large majority of patients, stapes surgery is associated with some risk. The risk for complete hearing loss on the operated ear is very low, on the order of 1%^5^. However, many studies have shown that the benefit from surgery declines with stimulus frequency and at 6 and 8 kHz, some loss of both air and bone conduction thresholds is commonly seen^6^,^7^,^8^,^9^. In two previous studies^10^,^11^, where the frequency range of audiometry was extended beyond the standard 8 kHz, a mean threshold loss ranging from 9 to 23 dB was evident across the frequencies 10–14 kHz, despite robust improvement of low-frequency hearing. The relevance of changes in hearing thresholds at these very high stimulus frequencies is unclear, although tinnitus patients often have elevated thresholds at 10 kHz and higher, which may correlate with the pitch of their tinnitus^12^.

Most patients with otosclerosis have tinnitus^13^, and many will experience improvement after surgery^14^. Previous studies however showed worsening of tinnitus in between 1 and 11% of patients^14^,^15^,^16^,^17^,^18^. Presently, there are no factors that allow surgeons to determine whether a patient is likely to experience increasing tinnitus, although some studies suggest that surgical techniques are important^16^.

A possible reason for the lack of improvement in high-frequency hearing and the increasing tinnitus experienced by some patients is that sensory cells are affected by surgical trauma, perilymph leakage, and sounds generated by drilling. Animal studies suggest that pharmacological intervention can target some of these factors. For instance, loud sound generates free radicals in the hearing organ^19^, and antioxidants such as N-Acetylcysteine therefore have significant protective effects in animals^20^,^21^. N-Acetylcysteine has also shown some effect against surgical trauma in animal models^22^. This compound was tested in two small clinical trials that targeted temporary threshold shifts^23^,^24^, and a small protective effect was found in one of the trials.

In the present study, we examined associations between high-frequency hearing (>8 kHz), tinnitus, and patient satisfaction in a cohort of 133 patients undergoing stapedotomy. These patients participated in a clinical study that examined whether N-Acetylcysteine has protective effects against permanent threshold shifts. The primary outcome measure in that trial^25^, was the hearing threshold at 6 and 8 kHz one year after surgery. Extended-frequency hearing measurements were included for exploratory purposes and form the basis of the present report, in addition to patient scores of tinnitus and satisfaction with surgery.

## Results

### Baseline patient characteristics

**Figure 1:**
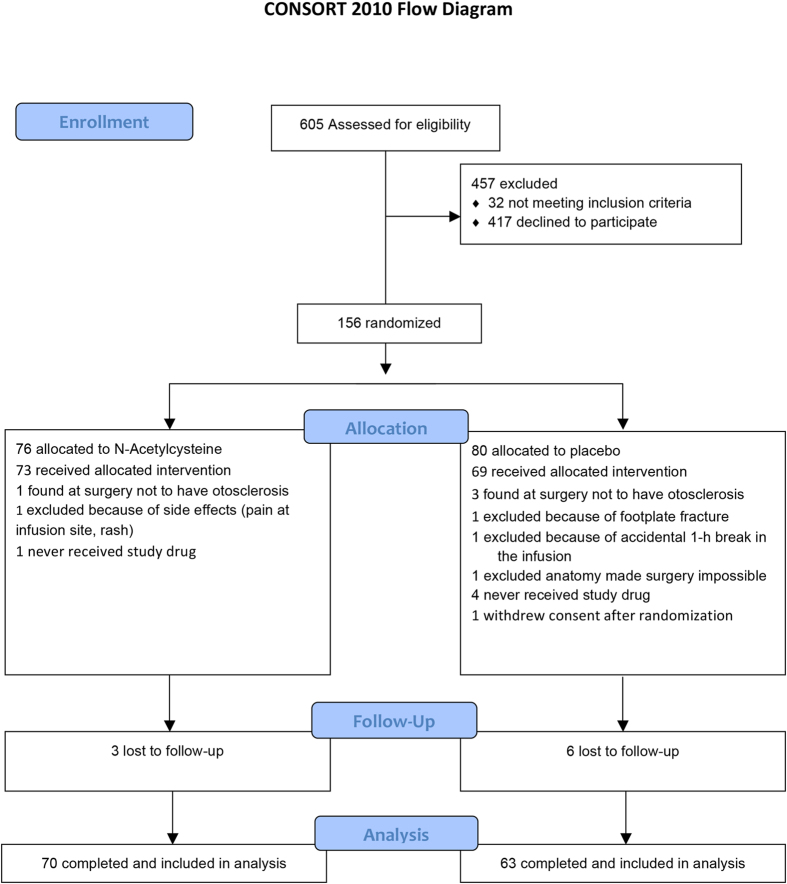
CONSORT flow diagram. Randomization took place before surgery. Patients returned for follow-up visits 6 to 8 weeks and one year after surgery. The numbers given refer to patients completing the entire study.

Starting in November 2007 and ending in April 2012, 605 patients with otosclerosis were assessed for eligibility (Fig. 1; the main eligibility criteria were otosclerosis where surgical treatment was planned, air-bone gap exceeding 20 dB across the frequencies 0.5 to 3 kHz, and age >18 years). From these potentially eligible subjects, 26% fulfilled inclusion criteria and were willing to participate in the trial. A common reason for declining participation was that patients lived far from the clinic and were unwilling to travel; some patients also declined to participate in high-frequency audiometry because of the extra time needed for hearing measurements. A total of 135 patients provided high-frequency audiometry data 6 weeks after surgery and 133 did so at one year (70 patients in the N-Acetylcysteine group, 63 in the placebo group).

**Figure 2:**
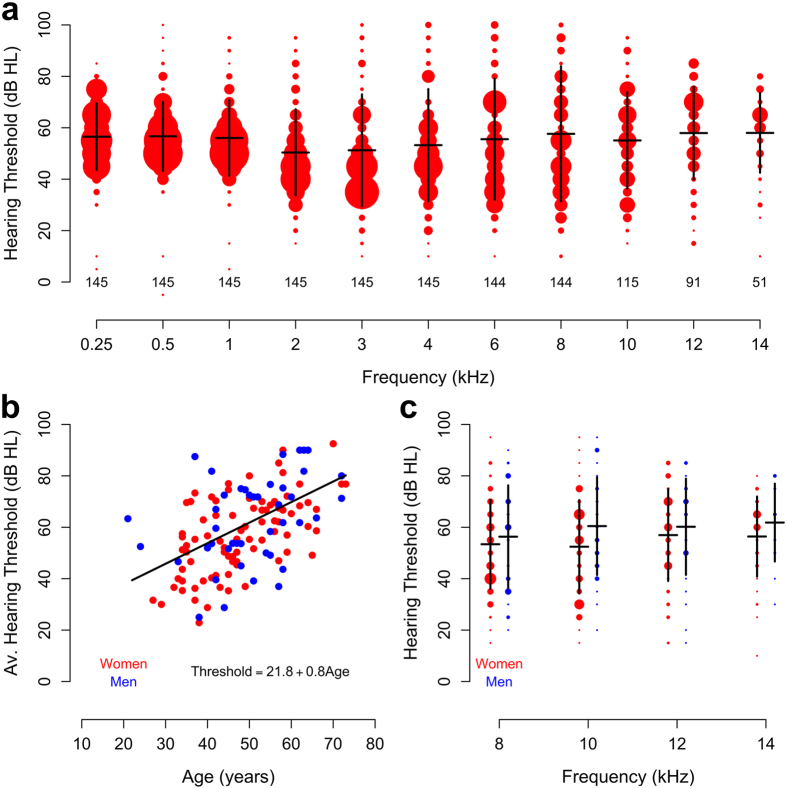
Patient characteristics before surgery. (**a**) Hearing thresholds for air-conducted sound. The size of each red dot corresponds to the number of patients, as determined from histograms with 5 dB bin widths. Horizontal black lines denote means, vertical lines the standard deviation. Numbers on the graph show the number of patients contributing to each average. High-frequency audiometry refers to the frequencies between 8 and 14 kHz. (**b**) Average thresholds for air-conducted sound across the frequencies 10 to 14 kHz as a function of patient age. (**c**) High-frequency air-conduction thresholds according to the sex of the patient. Red dots represent women, blue dots men. Dot sizes correspond to the number of patients at each threshold value (5-dB bins). Black lines denote means and standard deviations.

Patients in the placebo group were 48.1 ± 11 years old (mean ± standard deviation (sd)), which was not significantly different from the N-Acetylcysteine group (49.1 ± 11 years; p = 0.63, t-test). The majority of patients were female (69% in the placebo group vs. 63% in the N-Acetylcysteine group; p = 0.7; χ^2^-test) and most had poor high-frequency air conduction thresholds prior to surgery (Fig. 2a; this figure also includes thresholds for air-conducted sound at the conventional set of frequencies, 0.25–8 kHz). With the exception of the Carhart notch frequencies, the average thresholds show the flat loss near 60 dB that is typical for advanced otosclerosis. Before surgery, there were no significant differences in air conduction thresholds between the N-Acetylcysteine and the placebo group (p = 0.54, linear mixed model). As reported previously^25^, N-Acetylcysteine had no effect on tinnitus and in the present study we found that it had no significant effect on high-frequency hearing thresholds after surgery (p = 0.59, linear mixed model, see reference 25 for additional details regarding the conventional set of frequencies and further characteristics of patients and surgical factors). We therefore performed further analysis without separating patients on the basis of the drug given.

Before surgery, there was little difference in average air conduction thresholds between the conventional frequencies and those included in high-frequency audiometry (Fig. 2a; the frequencies included in each type of measurement are listed under methods), although it should be noted that many subjects had thresholds outside the range of the high-frequency audiometers. The number of subjects contributing to each average therefore decreases at frequencies higher than 8 kHz (as indicated by the numbers given on the graph). On average, hearing declined with frequency at a rate of 1.0 dB/kHz across the frequency range between 8 and 14 kHz (p < 0.001; linear mixed model).

Age was a strong predictor of pre-operative high-frequency hearing thresholds. To illustrate this, the average air conduction thresholds between 8 and 14 kHz was computed for each subject and plotted against age (Fig. 2b). Thresholds increased at a rate of 0.8 dB/year, an effect that was statistically significant (p < 0.001; linear mixed model). On average, women in this study had high-frequency hearing thresholds that were 5 dB better than men (Fig. 2c), but this difference was not statistically significant (p = 0.21, linear mixed model).

### Changes in high-frequency hearing after surgery

**Figure 3:**
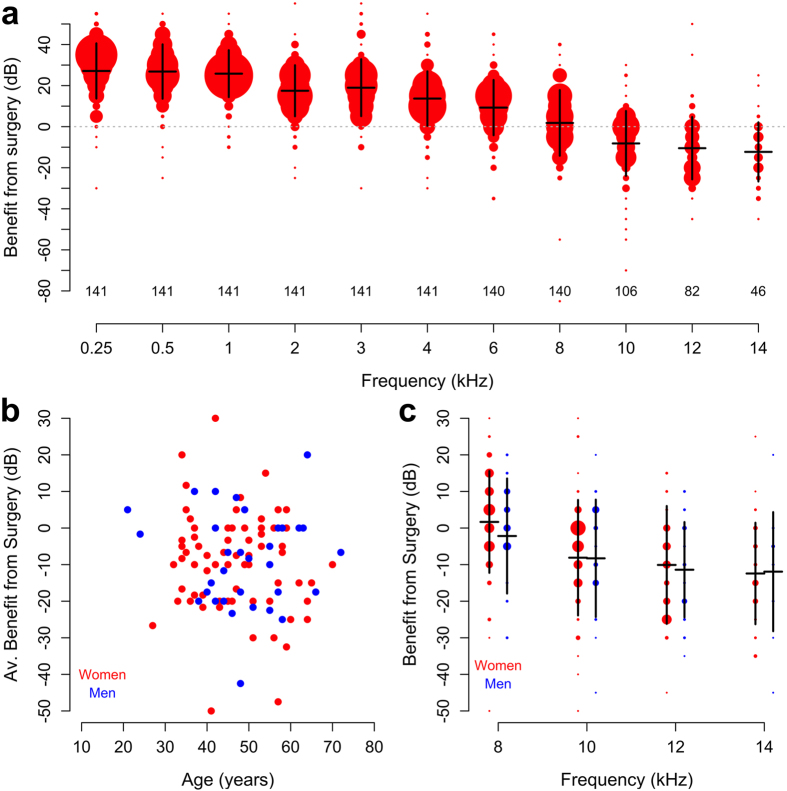
(**a**) Change in air conduction thresholds 6 weeks after surgery. As in Fig. 2, the size of the dots correspond to number of patients. Horizontal lines denote the mean; vertical lines represent one standard deviation. (**b**) Change in average hearing thresholds across the frequencies 10 to 14 kHz as a function of patient age. (**c**) Surgically-induced hearing changes according to patient sex. Means ± standard deviation; size of dots correspond to number of patients. Red represents women, blue dots men.

Surgery was associated with a large gain of hearing at low frequencies, but this benefit decreased with stimulus frequency (Fig. 3a). Hence, 80% of patients (115 out of 144) had measurable air conduction thresholds at 10 kHz before surgery, but only 74% had detectable thresholds after surgery. The fraction of patients with measurable hearing thresholds changed from 65% to 57% at 12 kHz, and from 36 to 33% at 14 kHz.

This postoperative decline in high-frequency hearing was reflected in the average thresholds of patients with measurable hearing on both occasions, where thresholds worsened by 8.2 ± 15.8 dB at 10 kHz, by 10.5 ± 15.2 dB at 12 kHz and by 12.3 ± 14.4 dB at 14 kHz (means ± standard deviation). The trend toward loss of hearing at higher frequencies after surgery was statistically significant (p < 0.001, linear mixed model, model slope−1.6 dB/kHz).

Although age was a significant predictor of absolute hearing thresholds before surgery (Fig. 2b), it had no influence on the change in high-frequency hearing caused by surgery (Fig. 3b; R^2^ for the correlation between the age of the patient and the average benefit from surgery across the frequencies 10–14 kHz was 0.01; p = 0.28). Also, there was no significant relation between the sex of the patient and the change in hearing thresholds 6 weeks after surgery (Fig. 3c).

Temporary effects on the cochlea or middle ear may affect hearing thresholds measured soon after surgery. Hence, we also examined hearing 1 year after the operation. At this point in time, 70% of patients had measurable air conduction threshold at 10 kHz. Corresponding figures at 12 and 14 kHz were 57 and 32%, respectively. In patients with measurable hearing thresholds both before and after surgery, the average loss was 5.8 ± 15.8 dB at 10 kHz, 10.3 ± 15.0 dB at 12 kHz and 9.4 ± 16.6 dB at 14 kHz at 1 year. Hence, in the time period between 6 weeks and one year, hearing improved by up to 3 dB in the range from 10 to 14 kHz, but this small change was not statistically significant.

We conclude that high-frequency hearing is adversely affected by stapes surgery. Only a limited recovery occurs during the first year, and the average patient will experience worsening of hearing thresholds at very high stimulus frequencies, despite the large and substantial improvement that occurs in the speech-frequency range.

### Tinnitus and changes in high-frequency hearing

**Figure 4:**
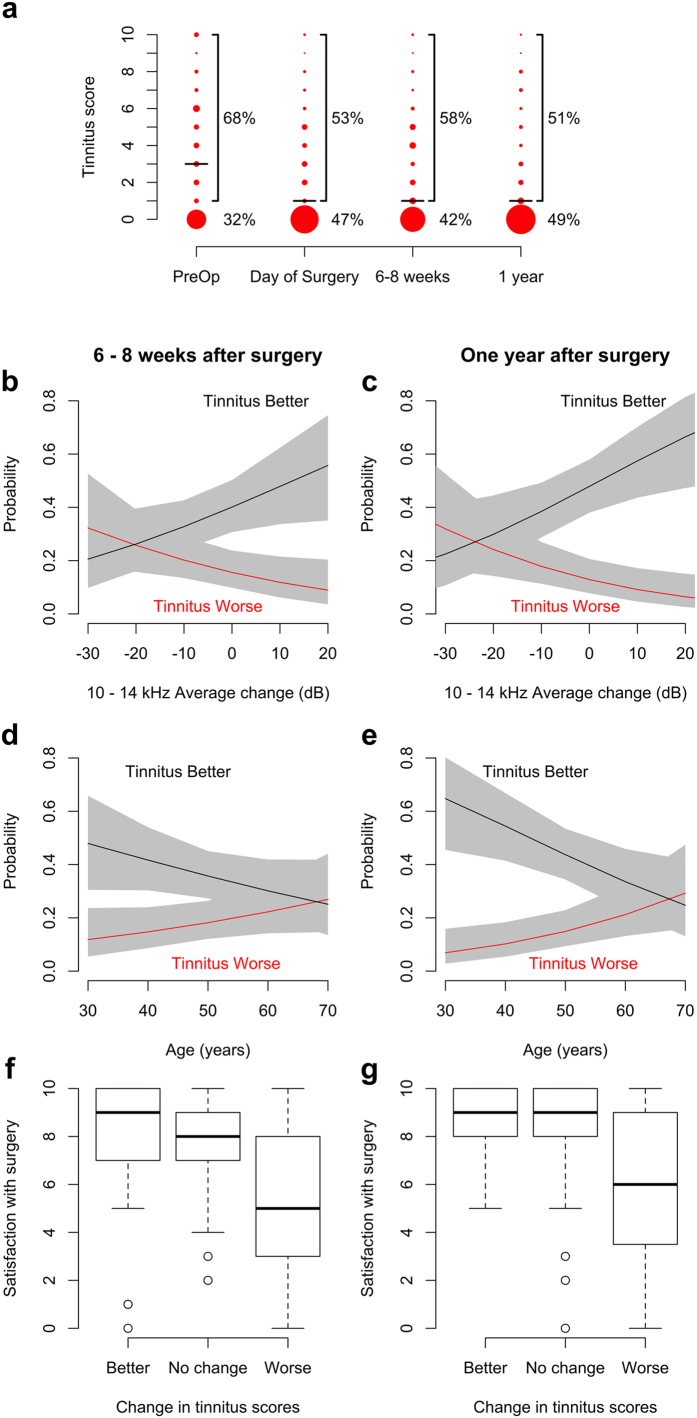
(**a**) Distribution of tinnitus scores before and after surgery. Dot sizes correspond to number of patients. Black horizontal lines denote the median values at each time point. (**b**) Proportional odds logistic regression models show how the probability of tinnitus becoming better depends on the average change in high frequency thresholds after surgery (black line). The probability of tinnitus becoming worse is plotted with the red line. Gray fields represent 95% confidence intervals. (**c**) Probability of tinnitus change at one year as a function of the average change in hearing thresholds across 10 to 14 kHz. (**d**) Relation between probability of tinnitus change and patient age at 6 weeks. (**e**) At one year, the probability of a change in tinnitus depends on age. (**f,g**) Patient satisfaction scores depend on the change in tinnitus scores at both 6 weeks and one year.

Patients graded their tinnitus on a 10-grade scale, where zero corresponded to no tinnitus and 10 to very disturbing tinnitus. Before surgery, 68% of patients reported tinnitus (Fig. 4a). Improvement occurred in many patients and in the evening on the day of surgery, 53% had tinnitus. This percentage increased slightly at 6 weeks (58%) but then fell to 51% at one year. The increased number of patients without tinnitus translated into a 2-unit decrease in median scores (from 3 to 1 on the 10-grade scale; the corresponding mean scores were 3.4 ± 3.3 and 2.4 ± 2.9). This change, the magnitude of which remained stable throughout the observation period, was statistically significant (p < 0.001; Wilcoxon signed rank test).

Despite the significant improvement in tinnitus scores, there were patients that reported worse tinnitus after surgery (overall, 19% gave a higher score immediately after surgery and at the 6-week visit, and 18% did so at one year. 23% of patients with a tinnitus score of 0 before surgery reported tinnitus immediately afterwards, but this fraction fell to 19% at both six weeks and one year). Proportional odds logistic regression models were used to explore factors that may contribute to this apparent variation. Six weeks after surgery, the average change in air conduction thresholds across the frequencies 10–14 kHz had significant (p = 0.04; Fig. 4b) influence on tinnitus scores. Patients with loss of high-frequency hearing had 32% probability of tinnitus going worse after surgery (and 21% probability of improvement). In contrast, patients with good high-frequency thresholds after surgery had 56% chance of improvement and only 9% probability of tinnitus becoming worse. These relations became stronger over time and at one year (Fig. 4c), a patient with substantially improved high-frequency air conduction thresholds had 74% chance of improvement in tinnitus scores and less than 5% risk of worsening (p < 0.01).

Patient age also influenced the change in tinnitus scores. Although the effect of age was not significant at 6 weeks (p = 0.12; Fig. 4d), it did become significant at 1 year (p = 0.01; Fig. 4e), where a 30-year old patient had 65% chance of improved tinnitus scores after surgery. One year after surgery, a patient at 70 years of age had higher probability of “no change” than improvement (46% probability of “no change” vs. 29% probability of worsening and 24% chance of improvement). Neither sex, body weight, nor the type of treatment given (N-Acetylcysteine or placebo) had significant influence on the change of tinnitus scores caused by surgery (all p values >0.1).

### Patient satisfaction

Patients used a 10-grade scale to score their satisfaction with surgery, zero corresponding to low and 10 to very high satisfaction. There was a clear relation between satisfaction scores and the change in tinnitus scores. Six weeks after the operation (Fig. 4f), patients experiencing improvement of tinnitus had a median satisfaction score of 9 (interquartile range (IQR), 2.75) whereas patients with worsening of tinnitus had median satisfaction score of 5 (IQR, 4.75). The relatively wide IQR means that some patients are satisfied with the outcome of the operation despite worsening tinnitus. The differences were statistically significant. Using proportional odds logistic regression, the likelihood of a satisfaction score higher than 7 was 81% in the group with improvement of tinnitus and 43% in the group with worsening of tinnitus (p < 0.01).

At one year (Fig. 4g), patients with tinnitus improvement continued to have a median satisfaction score of 9 but the IQR had decreased to 2. A small improvement was noted in the group with worsening of tinnitus (median score 6), but the IQR remained large (5.5), indicating substantial variability. A 94% likelihood of a high satisfaction score was found in patients with improved tinnitus; the group with worsening of tinnitus had 50% likelihood of scoring satisfaction higher than 7 (p < 0.001).

Neither age nor high-frequency thresholds had significant influence on patient satisfaction with surgery.

## Discussion

Our data shows that high-frequency hearing declines after stapes surgery, that young subjects have a high probability of improvement in tinnitus, but older patients and those with more pronounced loss of high-frequency hearing have a higher risk of worsening tinnitus. Hence, hearing at very high frequencies matters to many otosclerosis patients and efforts to further improve surgical results in this frequency range are warranted.

These findings have practical consequences for otosclerosis patients and their surgeons. Young patients can be advised that surgery is likely to improve their tinnitus, but a less positive message should be conveyed to older patients with a similar problem (Fig. 4d,e). Although age has significant influence on the chance of tinnitus improvement, age does not influence the gain in hearing thresholds seen after the operation. Thus, patients of all ages can expect meaningful improvement of hearing thresholds. This is important, since hearing loss is the accepted indication for performing surgery.

Why does stapedotomy improve tinnitus in many patients? Although the present data cannot provide a definitive answer, we note that the improvement was evident early, already in the evening on the day of surgery. This short interval likely does not allow for significant changes in the neural mechanisms^26^,^27^, thought to maintain tinnitus. However, Fig. 4b,c shows that tinnitus is more likely to improve in patients with good high-frequency hearing thresholds. Hence, more efficient acoustic masking is a possible explanation for the improvement in tinnitus seen after the operation. Patients with loss of high-frequency hearing after surgery are less likely to benefit from such masking effects and may also have suffered damage to sensory cells and neurons in the basal turn of the cochlea. Attention effects could also be important. In this case, the improved middle ear transmission would give patients a new acoustic environment that directs their attention away from tinnitus.

Previous studies typically show a 65% preoperative prevalence of tinnitus among patients with otosclerosis^13^, which is quite close to the value reported here (68%). All previous studies also show that surgery improves tinnitus in many patients, but the reported rate of deterioration varies widely (from 1.3% to 11%). The 18% rate reported here thus appears high, but it should be noted that scoring techniques differed among the published studies, making direct comparisons difficult, and that chance effects may influence results. For instance, Ayache *et al.*^17^ reported worsening postoperative tinnitus in 2%, corresponding to 1 out of 48 patients for whom follow-up data were available (26% of patients were lost to follow-up). A few extra patients with increasing tinnitus would have had a substantial effect. A much higher deterioration rate, 11%, was reported from the 246 stapedectomy patients studied by Ramsay *et al.*^14^, but this result may be influenced by recall bias, since patients answered questionnaires some time after surgery. Thus, the true rate of tinnitus deterioration after surgery remains unclear, but may be as high as 20%.

Two previous studies examined hearing thresholds in the 10–20 kHz frequency range after stapes surgery. Both included stapedectomies as well as stapedotomies. Mair and Laukli^10^ found an average threshold shift ranging from 15 to 22 dB between 10 and 14 kHz, while Tange and Dreschler^11^ showed a mean loss between 8.7 and 11.9 dB. Our results are in line with Tange and Dreschler’s, suggesting that surgical results at these frequencies have not improved substantially over the past 20 years. Other types of middle ear surgery may be associated with a small high-frequency hearing loss, but in these cases the loss is typically transient^28^.

The worsening of high frequency hearing^6^ signals a need for further development of surgical techniques and supportive care. Animal studies suggest that high-frequency sensory cells can be protected from surgical trauma^22^ and loud sounds^19^ by administration of antioxidants, such as N-Acetylcysteine^20^,^21^. Despite the effect evident in animal studies, this study and its companion^25^ do not support N-Acetylcysteine as an effective protective treatment in humans. The same conclusion is evident from a study of 566 noise-exposed recruits in the US Marine Corps, where a daily oral dose of 900 mg of N-Acetylcysteine failed to significantly influence hearing thresholds (except in exploratory analyses not part of the original study protocol)^29^. More potent antioxidants may have better effects, and other classes of drugs^30^, have also been shown to exert inner-ear protective effect in various animal models. Hence, further clinical trials are motivated in this area.

In conclusion, stapedotomy is associated with a large gain of hearing at low frequencies, but high-frequency hearing deteriorates in most patients. The number of patients free from tinnitus increased after surgery, remained stable at 1 year, and was more evident in younger patients. These patients tend to be very happy with the outcome of the operation, but patients that report worsening of tinnitus have significantly lower satisfaction scores.

## Methods

Patients in the present study participated in a clinical trial that examined whether a protective effect of antioxidants was present in patients undergoing stapes surgery. Extended-frequency audiometry was included as an exploratory measure of hearing function and these previously unreported data form the basis of the current study, together with patient assessments of tinnitus severity and satisfaction with surgery.

The original study was a randomized, double-blind, and placebo-controlled parallel-group trial (1:1 allocation ratio) performed at three Swedish university clinics. The protocol was approved by the Regional Ethics committee in Stockholm and by the Medical Products Agency. The trial was registered with Clinicaltrials.gov (accession number NCT00525551) and by EudraCT (2006-006243-31).

### Criteria for inclusion and exclusion

Patients were eligible for the study if they had signed the consent form, were older than 18 years of age, and had otosclerosis where surgical treatment was planned. The air-bone gap had to be at least 20 dB at 0.5, 1, 2 and 3 kHz; patients were required to have normal middle ear status without signs of infection or perforation of the eardrum. Due to a higher risk of side effects^31^, patients with asthma were excluded from participation, as were patients with known hypersensitivity to N-Acetylcysteine, only one functioning ear, and those with previous middle ear surgeries. In premenopausal women not using hormonal anticonception or intrauterine devices, serum chorionic gonadotropin was measured before administering the study drug; a positive test led to exclusion from the trial.

### Hearing assessment

Hearing was measured in soundproof booths with patients wearing calibrated headphones (HDA200 for high-frequency audiometry and TDH39 for conventional-frequency audiometry). First, pure tones at 0.25, 0.5, 1, 2, 3, 4, 6, and 8 kHz were presented to one ear at a time and thresholds for bone-conducted sound measured by placing a calibrated vibrator on the mastoid process while presenting tones at 0.25, 0.5, 1, 2, 3, and 4 kHz. Then, high-frequency audiometry was performed at the frequencies 8, 10, 12, 14 and 16 kHz. However, few subjects had measurable hearing at 16 kHz and this frequency was therefore excluded from analysis. The hearing threshold was identified using the modified Hughson-Westlake method as recommended by the International Standards Organization. The output of the audiometers was limited to the following values (all given in dB hearing level): 16 kHz, 60 dB; 14 kHz, 80 dB; 12 kHz, 90 dB; 10 kHz, 95 dB; 8 kHz, 100 dB.

In addition to hearing threshold measurements, patients graded tinnitus by answering the question “Have you been troubled by tinnitus (sounds in the ears) during the past week?”; possible answers ranged from zero (“Not at all”) to 10 (“To a high degree”). As formulated in Swedish, this question primarily measures annoyance with tinnitus.

These measures were performed before surgery, 6–8 weeks thereafter, and at one year. On the two last occasions, satisfaction with surgery was assessed with a 10-grade rating scale similar to the one used for tinnitus (“How do you rate the result of the operation?”, with answers ranging from “very poor” (0), to “very good” (10)).

### Surgery

Operations were performed by eight experienced otologic surgeons, who were free to choose the technique they deemed best. Hence, a laser was used in 35 patients for vaporizing the tendon and the posterior and anterior crus of the stapes, as well as for thinning the stapes footplate, which was then perforated with a microdrill. In all other cases, a microdrill was used throughout surgery. Ten percent of patients received general anesthesia in addition to infiltration of lidocaine-epinephrine; remaining patients received local anesthesia with lidocaine-epinephrine, supplemented with sedatives and anti-emetics. Additional details can be found in ref. 25.

### Randomisation

The techniques for stratified randomisation used in this trial are described in reference 25.

### Outcomes

High-frequency audiometry data (10 to 16 kHz) form the basis for the present report, together with patient scores of tinnitus severity and satisfaction with surgery, as detailed above.

### Statistical analysis

In clinical audiometry, hearing thresholds are repeatedly measured at different frequencies in each subject. This introduces correlations that need to be considered during statistical analysis. In addition, thresholds are determined at several different time points in each patient. To account for these autocorrelations, linear mixed models were used. The random effect in the model was a patient-dependent intercept, and the fixed effects were patient age, sex, stimulus frequency, and the medication given (placebo or N-Acetylcysteine). The dependent variable was the change in hearing thresholds after surgery. When baseline data were analyzed, the dependent variable was the absolute hearing threshold.

Patients scored tinnitus on a 10-grade scale, as described above. In most analysis, we subtracted postoperative from preoperative scores, resulting in a positive value in patients where tinnitus improved. To facilitate data presentation, we collapsed tinnitus scores into three categories, “worsening” for patients with negative scores, “Improvement” for those with positive scores, and “no change” for the remainder. This facilitates the presentation of results but had no influence on statistical significance testing. Data were analyzed with proportional odds logistic regression models in addition to the Wilcoxon signed rank test.

Calculations were performed using the lme and polr packages in R (v. 3.0.2; R Foundation for Statistical Computing 2013).

## Additional Information

**How to cite this article**: Bagger-Sjöbäck, D. *et al.* High-frequency hearing, tinnitus, and patient satisfaction with stapedotomy: A randomized prospective study. *Sci. Rep.* **5**, 13341; doi: 10.1038/srep13341 (2015).
